# Frecuencia de reabsorción intracoronal preeruptiva en radiografías panorámicas de pacientes peruanos entre 3 y 21 años. Un estudio transversal

**DOI:** 10.21142/2523-2754-1203-2024-207

**Published:** 2024-09-17

**Authors:** Iván Eduardo Pérez Lip, Carmen Rosa García Rupaya, Vilma Elizabeth Ruiz García de Chacón

**Affiliations:** 1 Universidad Peruana Cayetano Heredia. Lima, Perú. ivan.perez@upch.pe, carmen.garcia@upch.pe, vilma.ruiz.g@upch.pe Universidad Peruana Cayetano Heredia Universidad Peruana Cayetano Heredia Lima Peru ivan.perez@upch.pe carmen.garcia@upch.pe vilma.ruiz.g@upch.pe

**Keywords:** radiografía panorámica, anomalías dentarias, diente no erupcionado, germen dentario, intracoronal resorption, unerupted teeth, panoramic radiograph, tooth development

## Abstract

**Objetivo:**

Determinar la frecuencia de reabsorción intracoronal preeruptiva (RIPE) en una muestra de radiografías panorámicas digitales de pacientes peruanos entre 3 y 21 años que asistieron a un centro de radiología oral y maxilofacial entre los años 2013 y 2021.

**Materiales y métodos:**

El estudio observacional fue del tipo descriptivo, retrospectivo y transversal. Se evaluaron 1897 radiografías panorámicas digitales para determinar la frecuencia de la RIPE y sus covariables de localización, profundidad e inclinación de la pieza afectada en la muestra y en ambos sexos. La calibración de los autores se realizó antes de la ejecución del estudio, la concordancia intraoperador se determinó mediante el cálculo del coeficiente Kappa (0,72, IC 0,67-0,76), las radiografías fueron evaluadas por el autor principal (IEP) y los datos se recopilaron en una ficha diseñada para el estudio. La asociación estadística se determinó mediante la prueba de chi cuadrado con un nivel de confianza de 95% y un p-valor menor de 0.05 fue considerado como estadísticamente significativo.

**Resultados:**

La frecuencia de RIPE fue de 3,95% sin asociación significativa con respecto al sexo (p > 0,05), con respecto a las covariables, el segundo molar inferior derecho (32,5%), el defecto singular (89,3%), la localización central (44,5%) y la profundidad menor del tercio dentinal -grado I- (83,1%) fueron las más frecuentemente encontradas siendo estos resultados similares a los reportados en la literatura.

**Conclusiones:**

Según los resultados del presente estudio el perfil epidemiológico de la RIPE sería el de un defecto radiolúcido singular, ubicado en el centro de la dentina coronal próximo a la unión amelodentinaria, de tamaño pequeño (grado I), que afecta frecuentemente a piezas posteroinferiores en este orden: segundos molares, terceros molares y segundos premolares, y con una frecuencia menor del 5%.

## Abstract (en)

**Objective:**

To determine the frequency of Preeruptive Intracoronal Resorption (PIR) in a sample of digital panoramic radiographs of Peruvian patients between 3 to 21 years of age who attended an oral and maxillofacial radiology center between 2013 and 2021.

**Material and Methods:**

This observational study was descriptive, retrospective and cross-sectional. A sample of 1897 digital panoramic radiographs was evaluated to determine the frequency of PIR and its covariates of location, depth, and inclination of the affected teeth. The authors’ calibration was performed before the study. The intraoperator agreement was determined by calculating the Kappa coefficient (0.72, CI 0.67-0.76). The radiographs were evaluated by the study's principal author (IEP), and the data were collected in a form designed for the study. The statistical association was determined using the Chi-square test, with a confidence level of 95%, and a p-value of less than 0.05 was considered significant.

**Results:**

The frequency of PIR was 3.95%, with no statistically significant difference between sexes (p>0.05). The most common findings were PIR in the lower right second molar (32.5%), a singular defect (89.3%), central location (44.5%), and superficial dentinal involvement (83.1%). These results aligned with existing literature.

**Conclusions:**

Our results describe the epidemiological profile of PIR in the present sample as a single radiolucent and small (grade I) defect located centrally in the coronal dentin, often affecting posterior lower teeth with a frequency lesser than 5%. These findings provided valuable insights for clinicians and researchers regarding the frequency and characteristics of PIR in Peruvian patients.

## INTRODUCCIÓN

La reabsorción intracoronal preeruptiva (RIPE) consiste en el hallazgo radiográfico de un defecto radiolúcido semicircular ^1^, ubicado en la dentina coronal subyacente a la unión amelodentinaria de un germen dentario ^2^ que compromete entre ⅓ y ⅔ del ancho dentinal ^3^ y de una apariencia similar a la caries dental por lo que ha sido denominado como “caries preeruptiva”, “caries oculta” ^2^, “caries intrafolicular”, “lesión radiolúcida similar a la caries dental” o “resorción externa idiopática de piezas no erupcionadas” ^4^.

La etiología de la RIPE no ha sido determinada, pero se han propuesto distintas teorías: (a) condición producto de la inflamación pulpar de la pieza decidua, (b) secuela de la infección del germen dental por microorganismos cariogénicos, (c) el resultado de defectos de desarrollo en la dentina con o sin compromiso del esmalte dental, (d) una secuela de resorción externa o interna y (e) el producto de la posición ectópica del germen dental afectado o su vecino ^4^^,^^5^. La mayor frecuencia de piezas posteriores afectadas sin predecesores deciduos como segundos o terceros molares ^5^, y la nula evidencia de infección bacteriana en gérmenes dentarios ^6^ rechazan las dos primeras teorías. Además, en reportes y series de casos se han encontrado evidencias histológicas de lagunas de resorción y sus componentes celulares (células multinucleadas, macrófagos, osteoclastos y sustancia cementoide u osteoide) en los defectos de RIPE ^5^^,^^7^ ubicados en zonas de dentina de apariencia normal y, aunque no se ha determinado el activador ^5^, esta es la hipótesis más aceptada.

La detección y el control de las piezas afectadas solo se puede realizar mediante radiografías, y la radiografía panorámica es la técnica ideal para este propósito ^1^. La importancia clínica de la RIPE radica en el riesgo de superposición poseruptiva de la caries dental, debida a la colonización de microorganismos cariogénicos en el defecto dentinario existente ^8^^,^^9^.

En la literatura, la frecuencia de la RIPE varía entre un 0,5 y un 23,7%, y ha sido estudiada en muestras de radiografías panorámicas en distintos países: Arabia Saudita, Australia, China, Corea del Sur, Israel, Jordania, India, Tailandia y Turquía ^1^^,^^2^^,^^4^^,^^5^^,^^10^^-^^16^. No se han encontrado asociaciones con respecto al sexo, etnicidad o fluoración del agua, y tampoco se han reportado casos de piezas deciduas, debido a que no es frecuente indicar estudios radiográficos a pacientes menores de 3 años ^1^.

No se han realizado estudios descriptivos de la RIPE en el Perú o Latinoamérica y, por lo tanto, el objetivo del presente estudio fue determinar la frecuencia y distribución de la RIPE, así como sus covariables (localización anteroposterior, profundidad e inclinación del germen afectado) en una muestra de radiografías panorámicas de pacientes peruanos entre los 3 a 21 años que asistieron a un centro de radiología oral en la ciudad de Lima, Perú, entre los años 2013 y 2021.

## MATERIALES Y MÉTODOS

El presente estudio fue presentado al Comité de Ética de la Universidad Peruana Cayetano Heredia, con código 206932, y fue aprobado por exención. Solo el investigador principal (IEP) tuvo acceso a las imágenes radiográficas y en todo momento se mantuvo el anonimato de los pacientes.

El estudio fue observacional de tipo descriptivo y transversal fue realizado en 1897 radiografías panorámicas digitales de pacientes peruanos de 3 a 21 años que asistieron a un centro privado de radiología oral y maxilofacial ubicado en Lima, Perú, entre los años 2013 y 2021.

Las radiografías panorámicas digitales fueron adquiridas en un equipo de marca Planmeca, modelo Promax Scara 2 (Planmeca, Helsinki, Finlandia), los pacientes fueron posicionados según la técnica radiográfica estándar con valores de exposición de 70kV y 12 mA para pacientes adultos y 68kV y 12 mA para pacientes niños.

Las radiografías panorámicas digitales fueron visualizadas en el *software* radiológico Romexis, versión 4.6.2 (Planmeca, Helsinki, Finlandia) en un monitor Viewsonic VA2451 por el autor principal del estudio (IEP) en intervalos de descanso de 5 minutos por cada 30 minutos de evaluación ^17^. Los criterios de inclusión fueron (1) calidad radiográfica adecuada, (2) presencia de gérmenes dentarios posteriores en evolución intraósea a partir de corona completa (estadio 5 o 6 de Nolla), (3) presencia de gérmenes dentarios posteriores en evolución extraósea y con su corona en posición inferior con respecto a la unión cementoadamantina (UCA) de la pieza erupcionada contigua. 

**Figura 1:**
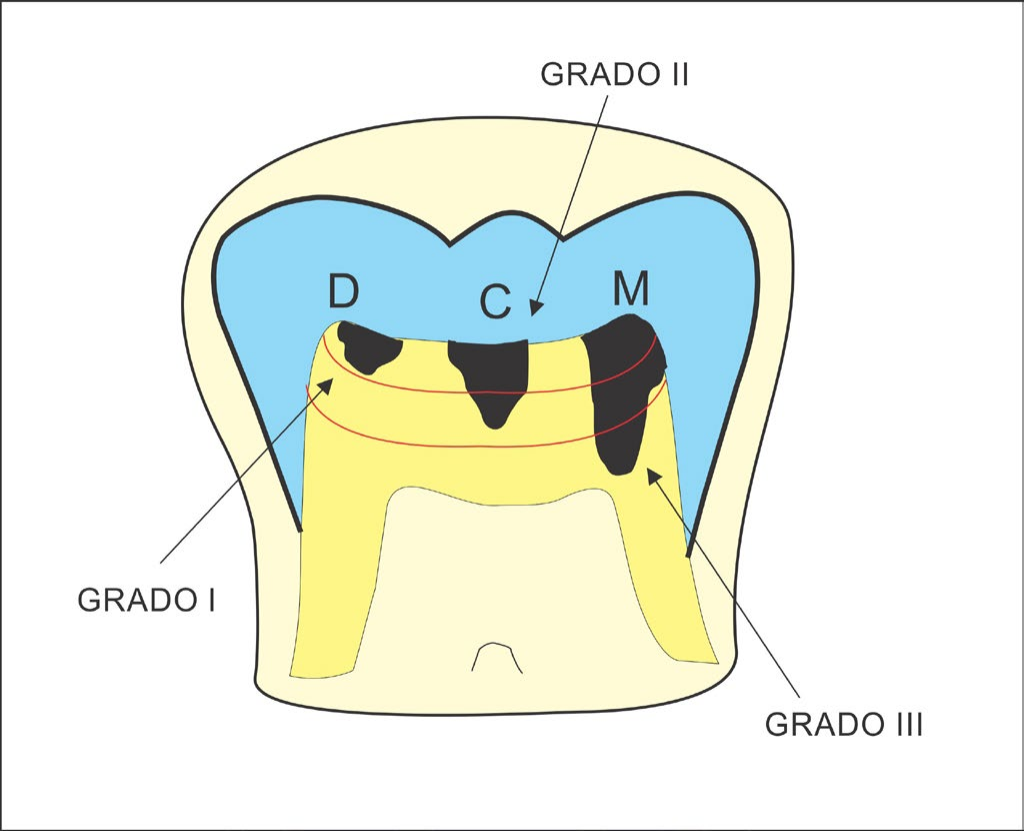
Primer molar inferior esquematizando de izquierda a derecha: posición distal (D), central (C) y mesial (M), y grados de profundidad I, II y II respectivamente. Fuente: autor.

**Figura 2:**
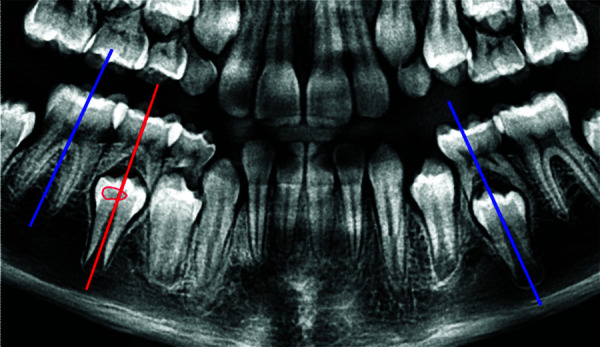
Imagen de radiografía panorámica que demuestra la comparación de la inclinación del eje vertical de la pieza 45 con RIPE (rojo) respecto a la pieza erupcionada vecina (46) y a la pieza contralateral (35).

Cada radiografía panorámica fue evaluada en busca de defectos compatibles con reabsorción intracoronal preeruptiva (RIPE), se utilizó la siguiente definición de variables:

1. RIPE, presencia de imagen radiolúcida de profundidad variable ubicada en la dentina coronal subyacente a la unión amelodentinaria (Fig. 1).

2. Localización anteroposterior, descripción de la posición de la lesión de RIPE según la división en tercios de la corona dentaria (mesial, centro y distal), según lo descrito por Seow *et al*. ^12^ (Fig. 1). 

3. Profundidad, valoración de la profundidad del defecto dentario de acuerdo con la división en tercios descrita por Seow *et al*. ^12^ (Fig. 1).

4. Inclinación de la pieza afectada, valoración del paralelismo del eje vertical de la pieza afectada con respecto a la pieza erupcionada vecina y a la pieza contralateral, según lo descrito por Seow *et al*. ^12^ (Fig. 2).

La calibración de los autores (IEP y VER) se realizó antes de la ejecución del estudio. La concordancia intraoperador se calculó mediante el coeficiente Kappa obtenido al comparar la evaluación, separada por dos semanas, de 350 radiografías panorámicas seleccionadas al azar de la base de datos y que no fueron incluidas en la muestra del estudio. El valor encontrado fue de 0,72 (IC 0,67-0,76) el cual se interpreta como una reproductibilidad sustancial.

### Plan de análisis

La información de las radiografías panorámicas fue recopilada en una ficha en formato MS Excel 2010. Se desarrollaron tablas de frecuencia con valores absolutos y relativos para la estadística descriptiva (promedio de edad, desviación estándar y percentiles), así como para las variables relacionadas a la RIPE (frecuencia, número de defectos, posición anteroposterior, profundidad del defecto e inclinación del germen afectado).

La asociación entre las variables fue determinada utilizando el *software* Stata versión 14 (StataCorp, TX, Estados Unidos) y mediante el cálculo del estadístico chi-cuadrado con un el nivel de confianza del 95% y un valor de “p” menor de 0,05 fue considerado como significativo. 

## RESULTADOS

Se evaluaron 1897 radiografías panorámicas digitales de pacientes entre los 3 a 21 años (57,5% femeninos y 42,5% masculinos) con un promedio de edad de 11,42 ± 3,92 años (11,59 ± 4,1 años en el sexo femenino y 11,20 ± 3,66 en el masculino) (Tabla 1).

**Tabla 1:** Estadística descriptiva de la muestra estudiada

	Mujeres	Hombres	Total
N.o de radiografías	1092	805	1897
Edad X_	11,59	11,2	11,42
Desv. estándar	4,1	3,66	3,92
Percentil 25	8	8	8
Percentil 50	12	11	11
Percentil 75	15	14	14

**Figura 3:**
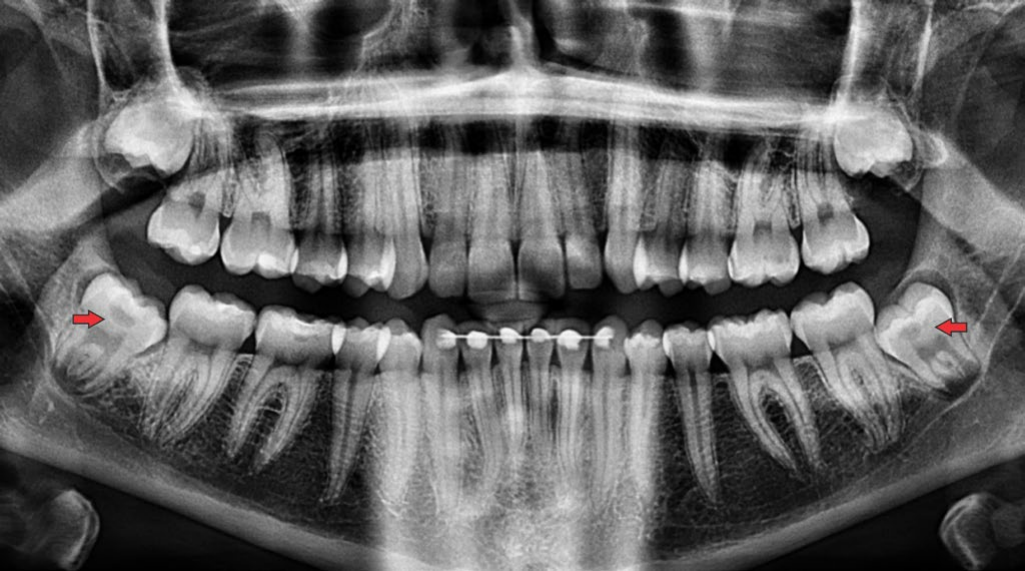
Imagen de radiografía panorámica en donde se indican (flecha roja) dos defectos de RIPE en las piezas 38 y 48.

Se encontraron 75 pacientes con RIPE (3,95%), de los cuales 49 pacientes fueron del sexo femenino (4,48%) y 26 pacientes del masculino (3,22%). Se encontró un total de 83 defectos en los 75 pacientes, en 67 pacientes se encontró un defecto (89,3%, 63% en el sexo femenino y 37 % en el masculino) y en 8 pacientes se encontraron dos defectos (10,7%, 88% en el sexo femenino y 12% en el masculino) (Tabla 2) (Fig. 3).

**Tabla 2:** Resumen de las variables estudiadas de la reabsorción intracoronal preeruptiva

	Femenino	%	Masculino	%	TOTAL	%
RIPE	49	4,48	26	3,22	75	3,95
No-RIPE	1043	95,52	779	96,78	1822	96,05
Total	1092	100	805	100	1897	100
Numero de defectos						
1 defecto	42	85,7	25	96,2	67	89,3
2 defectos	7	14,3	1	3,8	8	10,7
Total	56	67,5	27	32,5	83	100
Localización						
Mesial	18	32,1	4	14,8	22	26,5
Central	25	44,6	12	44,4	37	44,6
Distal	13	23,2	11	40,7	24	28,9
Profundidad						
Grado I	47	83,9	22	81,5	69	83,1
Grado II	4	7,1	4	14,8	8	9,6
Grado III	5	8,9	1	3,7	6	7,2
Inclinación / pza. vecina						
Presenta	31	55,4	12	44,4	43	51,8
No-presenta	25	44,6	15	55,6	40	48,2
Inclinación / pza. contralateral						
Presenta	11	19,6	0	0	11	13,3
No presenta	45	80,4	27	100	72	86,7

**Figura 4:**
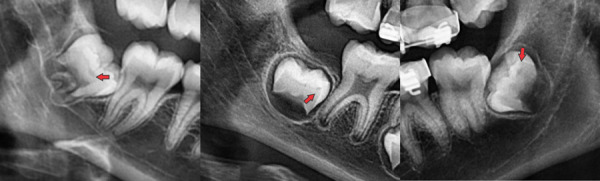
Secciones de radiografías panorámicas en donde se indican (flechas rojas) de izquierda a derecha defectos de RIPE en piezas 48 y 38 y en zonas central, mesial y distal respectivamente.

La localización anteroposterior más frecuentemente fue la central (44.6%), seguida de las localizaciones distal y mesial (28,9% y 26,5%), esta distribución fue similar en el sexo masculino mientras que en el femenino la distribución fue central/mesial/distal (Tabla 2) (Fig. 4).

**Figura 5:**
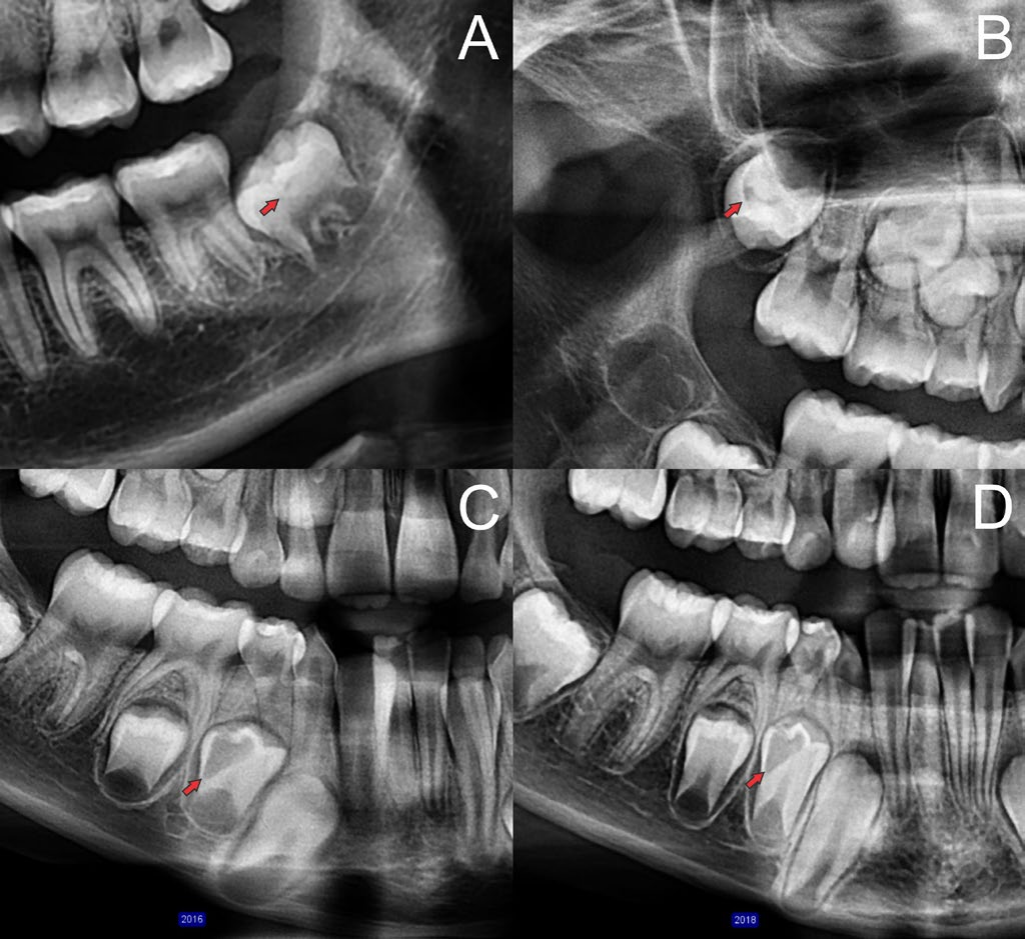
Secciones de radiografías panorámicas en donde se indican (flechas rojas) los distintos grados de profundidad de los defectos de RIPE (A) pieza 38, grado I; (B) pieza 18, grado II; (C y D) pieza 44 grado III tomadas con dos años de separación.

El grado I de profundidad fue el más frecuente (83,1%), seguido por los grados II y III (9,6% y 7,3%) en la muestra y el sexo masculino; en el sexo femenino, la distribución de los grados de profundidad fue I, III y II respectivamente (Tabla 2) (Fig. 5).

La frecuencia de inclinación del eje vertical de la pieza afectada respecto a la pieza vecina erupcionada fue de 51,8% en la muestra (55,4% en el sexo femenino y 44,4% en el masculino); mientras que la inclinación de la pieza afectada respecto a su contralateral fue del 86,7% (80,4% en el sexo femenino y 100% en el sexo masculino) (Tabla 2).

Se compararon las inclinaciones del eje vertical de las piezas con RIPE con respecto a las piezas vecina y contralateral, encontrándose una frecuencia de inclinado/no-inclinado del 45,8% y el 54,2% con respecto a la pieza vecina, y del 13,3% y el 86,7% con respecto a la contralateral, siendo esas diferencias estadísticamente significativas (p < 0,05) (Tabla 3).

**Tabla 3:** Comparación entre la inclinación de la pieza afectada respecto de las piezas vecina y contralateral

	Inclinación contra lateral-Si		Inclinación contralateral -No		Total	
	n	%	n	%	n	%
Inclinación del diente vecino- Si	10	90,9	33	45,8	43	51.8
Inclinación del diente vecino- No	1	9,1	39	54,2	40	48.2
Total	11	13,3	72	86,7	83	

Prueba exacta de Fisher, p < 0,05

Las piezas inferiores fueron significativamente las más frecuentemente afectadas en una relación de 3,75 a 1 (p < 0,05) (Tabla 4) y las piezas más frecuentemente afectadas fueron los segundos molares inferiores (32,5%), terceros molares inferiores (18,1%), segundos premolares inferiores (16,9%) y segundos molares superiores (12%); en el sexo femenino, la distribución de frecuencias fue similar mientras que en el masculino las piezas más frecuentemente afectadas fueron los segundos molares superiores (29,7%), segundos molares inferiores (22,2%), segundos premolares inferiores y terceros molares superiores (14,8% cada uno) (Tabla 5).

**Tabla 4:** Frecuencia de las piezas afectadas con RIPE con respecto al sexo y al tipo de maxilar

	Maxilar		Mandíbula		TOTAL*
	n	%	n	%	
Femenino	7	12,5	49	87,5	56
Masculino	13	48,2	14	51,9	27
	20	24,1	63	75,9	83

X2 = 12,6571 p < 0,05

* Se contabilizaron todos los defectos de los 75 pacientes.

**Tabla 5:** Frecuencia de las piezas posteriores más frecuentemente afectadas por la RIPE

	Femenino		Masculino		Total*	
	n	%^a^	n	%^a^	n	%
Segundos molares inferiores	21	37,5	6	22,2	27	32,5
Terceros molares inferiores	13	23,2	2	7,4	15	18,1
Segundos premolares inferiores	10	17,9	4	14,8	14	16,9
Segundos molares superiores	2	3,6	8	29,7	10	12
Terceros molares superiores	4	7,1	4	14,8	8	9,6
Primeros premolares inferiores	4	7,1	2	7,4	6	7,2
Segundos premolares superiores	1	1,8	1	3,7	2	2,4
Primeros molares inferiores	1	1,8	0	0	1	1,2
Total	56		27		83	

* Se contabilizaron todos los defectos de los 75 pacientes.

a Los valores corresponden a la frecuencia dentro del mismo sexo.

## DISCUSIÓN

El presente estudio fue realizado en una muestra de 1897 radiografías panorámicas de pacientes peruanos entre los 3 y 21 años. El diseño del estudio fue descriptivo, retrospectivo, transversal y de planteamiento similar a los estudios realizados desde 1999, cuando se publicó el primer estudio descriptivo de la RIPE por Seow *et al*. ^12^ en el que se definieron y clasificaron las variables de localización y profundidad de los defectos, tales clasificaciones se han seguido utilizando sin modificaciones en estudios posteriores ^1^^-^^16^.

La frecuencia encontrada de RIPE fue del 3,95%, sin asociación significativa con respecto al sexo, resultados similares y menores al 5% fueron reportados en varios estudios ^1^^,^^2^^,^^4^^,^^10^^-^^13^^,^^15^^,^^16^^,^^18^, mientras que frecuencias mayores fueron reportadas en cuatro estudios ^5^^,^^14^^,^^19^^,^^20^. Las diferencias entre las frecuencias reportadas podrían deberse a características propias de la RIPE en las poblaciones estudiadas, al tamaño de la muestra y a los rangos de edades. En la revisión de los casos reportados por Özden ^2^ y Uzun ^4^ se encontró que todos los casos pertenecieron a pacientes en el rango de 20 a 70 años, razón por la cual no es posible la comparación de resultados y podríamos considerar sus resultados como la frecuencia de reabsorción dentaria en piezas incluidas. En el estudio de Wang ^13^, la frecuencia reportada (0,85%) podría estar relacionada al menor tamaño de muestra o, también, al promedio de edades (9,3 años se sexo femenino y 9,4 años de masculino) que no incluiría a los terceros molares por no estar completamente desarrollados. En la literatura, la frecuencia reportada de la RIPE es menor al 10% y, salvo por los estudios de Konde ^14^ (13,6%) y Nik (20) (27,3%), podríamos concluir que la RIPE es una condición de baja frecuencia en la población estudiada.

La RIPE en el 89,7% de los casos se presentó como un defecto singular, nuestros resultados están en el rango de 88-100% encontrado en la literatura ^1^^,^^5^^,^^10^^,^^13^^-^^16^^,^^20^ y son mayores a los reportados por Seow ^12^^,^^19^. Esta preponderancia del defecto singular podría sugerir como causa a factores locales, tal y como se ha planteado en la literatura ^12^^,^^16^.

La localización central de la RIPE fue la más frecuentemente encontrada (44,6%), lo que concuerda con los resultados de cuatro estudios ^1^^,^^12^^,^^15^^,^^16^. Las localizaciones distal y mesial fueron la segunda y tercera más frecuentes en la muestra estudiada (28,9% y 26,5%), y en el sexo masculino (40,7% y 14,8%), mientras que, en el sexo femenino, la distribución fue mesial y distal (32,2% y 23,2%). La posición mesial fue la más frecuentemente reportada en cinco estudios ^5^^,^^10^^,^^14^^,^^20^ y la posición distal fue la más frecuentemente reportada en dos estudios ^13^^,^^18^. Debido a la variabilidad de la posición de los defectos reportada en la literatura ^1^^,^^5^^,^^10^^-^^16^^,^^18^^,^^20^ los hallazgos del presente estudio podrían corresponder a una característica propia de la muestra estudiada, la cual no es significativa según la evaluación estadística.

El grado de profundidad del defecto de RIPE más frecuentemente encontrado fue el grado I (83,1%), y resultados similares fueron reportados en la mayoría de los estudios ^1^^,^^5^^,^^10^^,^^13^^,^^14^^,^^16^^,^^18^^-^^20^. La mayor frecuencia de profundidad grado II y grado III fueron reportadas en dos estudios ^15^^,^^12^. En el sexo masculino, la distribución del grado de profundidad fue I, II y III (81,5%, 14,8% y 3,7%), mientras que en el sexo femenino la distribución fue de I, III y II (83,9%, 8,9% y 7,2%). Nuestros resultados coinciden con lo reportado en la literatura y, aunque no hay estudios de seguimiento, la profundidad más frecuentemente encontrada corresponde a la de un defecto de pequeñas dimensiones, lo cual es importante en el momento del pronóstico y planificación del tratamiento. 

La inclinación de la piezas afectada respecto a la pieza erupcionada vecina fue del 51,8% y del 13,3% respecto de la pieza contralateral, asociación significativa para lo que podría interpretarse como inclinación fisiológica del germen dentario afectado y no patológica, como se reportó en la literatura. Esta comparación de la inclinación de la pieza afectada con respecto a la contralateral no ha sido planteada en la literatura y podría ser considerada como un aporte al estudio de la RIPE. 

Las piezas más frecuentemente afectadas por RIPE fueron los segundos molares inferiores (32,5%), terceros molares inferiores (18,1%), segundos premolares inferiores (16,9%) y segundos molares superiores. En el sexo masculino, el orden de frecuencias en el sexo masculino fue de segundos molares superiores, segundos molares inferiores y segundo premolar inferior (29,7%, 22,2% y 14,8%, respectivamente), mientras en el sexo femenino fueron los segundos molares inferiores, terceros molares inferiores y segundos premolares inferiores (37,5%, 23,2% y 17,9%, respectivamente). En la revisión de la literatura, un estudio reportó como más frecuente al segundo molar mandibular ^1^, mientras que el primer premolar mandibular fue reportado como más frecuente en tres estudios ^5^^,^^14^^,^^20^, el primer molar mandibular fue la pieza más frecuentemente afectada en tres estudios ^12^^,^^16^^,^^19^, y en dos estudios se reportaron a las piezas mandibulares como las más frecuentemente afectadas ^15^^,^^18^.

En el presente estudio se encontró una preponderancia significativa de piezas inferiores (p < 0,05) con un factor de 3,5 a 1 con respecto a las piezas superiores. Resultados similares se han encontrado en más de la mitad de las referencias ^1^^,^^5^^,^^11^^,^^16^^,^^19^^,^^20^, mientras que en los estudios restantes las piezas más frecuentemente afectadas fueron los molares maxilares ^12^ y caninos ^15^^,^^18^. En la literatura se sugiere que la preponderancia de piezas inferiores podría estar relacionada a la influencia de la inclinación vestibulolingual fisiológica de las piezas posterosuperiores en el mecanismo de formación de la imagen panorámica ^5^^,^^12^^,^^13^^,^^16^^,^^19^^,^^21^, lo que determinaría la subestimación sistemática de la frecuencia de la RIPE. El uso de la TCHC nos permite visualizar, sin proyección de estructuras anatómicas, de todas las superficies dentales. Demirtas ^22^ comparó la frecuencia de RIPE en radiografías panorámicas y TCHC, y encontró una diferencia significativamente mayor de casos en TCHC (9,5%) respecto de la radiografía panorámica (3,1%). El presente estudio fue realizado en radiografías panorámicas las que, a pesar de las limitaciones, constituyen una técnica radiográfica ampliamente difundida, de leve exposición a la radiación y que permite reunir un considerable número de observaciones que aseguren la robustez del estudio.

Actualmente, la hipótesis más aceptada propone que la RIPE es una secuela de resorción de tejido dental calcificado ^5^ resultante de la invasión de osteoclastos, células gigantes multinucleadas y células inflamatorias crónicas ^23^^,^^24^, lo cual ha sido confirmado en reportes de caso por medio de la inmunorreactividad positiva a la catepsina-K en los defectos ^7^ y, aunque no se han determinado a los activadores, se considera como la más probable a la pérdida de integridad del epitelio protector ^5^. Se ha encontrado en estudios histológicos de terceros molares impactados ^25^ la presencia de metaplasia del epitelio reducido del órgano del esmalte del tipo cuboidal delgado con tejido conectivo laxo a escamoso estratificado plano no queratinizado con cambios inflamatorios del tejido conectivo circundante, tales cambios podrían confirmarse en los reportes de caso como una posible causa de migración de células inflamatorias a la dentina.

Aunque no se han desarrollado guías de práctica clínica que aborden el tratamiento de la RIPE, el conocimiento de la existencia de estos defectos permitirá al profesional el diagnóstico radiográfico oportuno, la valoración del pronóstico de la pieza afectada en considerando la presencia de una cavidad dentinaria con túbulos permeables ^26^ y separada de la cavidad bucal por una capa de esmalte sin soporte y amortiguación dentinaria ^24^, y el momento apropiado de la intervención clínica. 

### Limitaciones

La principal limitación del estudio fue que se ejecutó en radiografías panorámicas dentales (2D), las que no muestran con claridad, debido a la superposición de estructuras e inclinación dental fisiológica, a las piezas posterosuperiores y lo que supondría una subestimación de la frecuencia real de hasta 6% ^22^.

## CONCLUSIONES

La frecuencia de RIPE encontrada en la muestra de radiografías panorámicas evaluadas fue del 3,95% y se presentó como un defecto radiolúcido singular, en el centro de la dentina coronal, con una profundidad menor de 1/3 del ancho dentinal (grado I) y que se encontró en a piezas posteroinferiores: segundos molares, terceros molares y segundos premolares. No se encontró asociación de la frecuencia de RIPE con respecto al sexo y la inclinación de la pieza afectada por RIPE y su contralateral son significativamente similares, por lo que podría considerarse como fisiológica.

## Financiamiento:

Estudio autofinanciado
